# Tailoring Dzyaloshinskii–Moriya interaction in a transition metal dichalcogenide by dual-intercalation

**DOI:** 10.1038/s41467-021-23658-z

**Published:** 2021-06-15

**Authors:** Guolin Zheng, Maoyuan Wang, Xiangde Zhu, Cheng Tan, Jie Wang, Sultan Albarakati, Nuriyah Aloufi, Meri Algarni, Lawrence Farrar, Min Wu, Yugui Yao, Mingliang Tian, Jianhui Zhou, Lan Wang

**Affiliations:** 1 School of Science, RMIT University, Melbourne, VIC 3001 Australia; 2 Centre for Quantum Physics, Key Laboratory of Advanced Optoelectronic Quantum Architecture and Measurement (MOE), School of Physics, Beijing Institute of Technology, Beijing, 100081 China; 3 Beijing Key Lab of Nanophotonics & Ultrafine Optoelectronic Systems, School of Physics, Beijing Institute of Technology, Beijing, 100081 China; 4 International Center for Quantum Materials, School of Physics, Peking University, Beijing, 100871 China; 5 Anhui Province Key Laboratory of Condensed Matter Physics at Extreme Conditions, High Magnetic Field Laboratory, HFIPS, Chinese Academy of Sciences (CAS), Hefei, 230031 Anhui China; 6 University of Science and Technology of China, Hefei, 230026 Anhui China; 7 Department of Physics, School of Physics and Materials Science, Anhui University, Hefei, 230601 Anhui China; 8 Collaborative Innovation Center of Advanced Microstructures, Nanjing University, Nanjing, 210093 China

**Keywords:** Materials for devices, Magnetic properties and materials

## Abstract

Dzyaloshinskii–Moriya interaction (DMI) is vital to form various chiral spin textures, novel behaviors of magnons and permits their potential applications in energy-efficient spintronic devices. Here, we realize a sizable bulk DMI in a transition metal dichalcogenide (TMD) 2H-TaS_2_ by intercalating Fe atoms, which form the chiral supercells with broken spatial inversion symmetry and also act as the source of magnetic orderings. Using a newly developed protonic gate technology, gate-controlled protons intercalation could further change the carrier density and intensely tune DMI via the Ruderman–Kittel–Kasuya–Yosida mechanism. The resultant giant topological Hall resistivity ${\rho }_{{xy}}^{T}$ of $1.41{\mathrm{\mu}} \Omega \cdot {{\mathrm{cm}}}$ at ${V}_{g}=-5.2{\mathrm{V}}$ (about $424 \%$ larger than the zero-bias value) is larger than most known chiral magnets. Theoretical analysis indicates that such a large topological Hall effect originates from the two-dimensional Bloch-type chiral spin textures stabilized by DMI, while the large anomalous Hall effect comes from the gapped Dirac nodal lines by spin–orbit interaction. Dual-intercalation in 2H-TaS_2_ provides a model system to reveal the nature of DMI in the large family of TMDs and a promising way of gate tuning of DMI, which further enables an electrical control of the chiral spin textures and related electromagnetic phenomena.

## Abstract (web summary)

The complex spin textures induced by the Dzyaloshinskii–Moriya interaction (DMI) allow for a host of intriguing phenomena, however the strength of the DMI is typically fixed. Here, Zheng et al. demonstrate the controlled modulation of the DMI, via solid state ionic gating of a transition metal dichalcogenide.

## Introduction

The marriage of the broken local spatial inversion symmetry (SIS) and strong spin–orbit coupling (SOC) in magnetic materials could lead to the asymmetric exchange interaction, Dzyaloshinskii–Moriya interaction (DMI)^1,2^. DMI has attracted increasing attention due to its capability to stabilize the chiral spin textures, such as magnetic skyrmions, chiral domain walls^3–5^, and realize the novel physics of elementary excitations in magnetic insulators, including the spin Nernst Hall effect^6^, the thermal Hall effect of magnons^7,8^. Passing through the chiral spin textures, electrons can feel an artificial gauge field and accumulate a finite real-space Berry phase^9^, resulting in a remarkable transport signature-topological Hall effect (THE)^10,11^, which acts as the probe of chiral spin textures and the underlying DMI. Thus controlling DMI would greatly facilitate the manipulation of chiral spin textures and the related anomalous electromagnetic responses as well as their potential application in energy-efficient spintronic devices. DMI in bulk materials usually originates from the inversion asymmetry in the natural unit cells of crystals^12–14^, or the structural inhomogeneity along the thickness direction, as in amorphous ferrimagnets GdFeCo^15^. In contrast, realizing DMI in layered materials permits the manipulation of chiral spin textures and investigation of various related fascinating physics in few atomic layers. While in a large family of layered materials, SIS is respected in the natural unit cell and usually forbids DMI, we, however, demonstrate a promising way to induce sizable DMI by breaking SIS in the enlarged supercell through alternatively intercalating heavy magnetic atoms also as the source of magnetic orderings.

The intercalation of magnetic atoms (V, Cr, Mn, Fe, Co, Ni) in transition metal dichalcogenides (TMDs) 2H-TaS_2_ and -NbS_2_ leads to various magnetic ground states (including easy axis/plane ferromagnetism (FM) and antiferromagnetism (AFM))^16,17^ and novel strongly correlated states^18^, providing a leading-edge field searching for DMI and potential chiral magnetic structures. It has been reported that magnetic fields could turn the magnetically intercalated TMDs into novel chiral spin textures, such as the chiral solitons and chiral conical states in Cr_1/3_NbS_2_^19,20^, the chiral domain walls in Fe_1/3_TaS_2_^21^. Although DMI is crucial to these fascinating experiments above, the existence and nature of DMI in magnetically intercalated TMDs were, however, unknown. In addition, DMI in itinerate intercalated TMDs is usually dominated by the Ruderman–Kittel–Kasuya–Yosida (RKKY) interaction as the FM interactions^22^ and can in principle be controlled by tuning the carrier density. However, an electrical tuning of DMI so as to electrically control all the chiral spin textures in itinerate magnets is still a big challenge. This is because, for conventional gate technology, electric-field tends to be greatly screened in itinerant magnets by mobile carriers, which cannot effectively modulate the carrier density and FM. Ionic liquid (Li^+^)^23^ can accumulate substantial charge carriers. However, it can only tune the carriers close to the surface^24^.

In this article, we demonstrate that DMI can be induced and controlled in a TMD 2H-TaS_2_ by dual-intercalation. Intercalating Fe atoms into 2H-TaS_2_, Fe_1/3-δ_TaS_2_ (${\delta }\le 0.05$), DMI is confirmed by the observation of THE at low temperatures. Moreover, proton intercalation induced by electrical gating could further change the carrier density then largely tune DMI via the RKKY mechanism, resulting in a huge topological Hall resistivity of $1.4{\mu }\Omega \cdot {{\mathrm{cm}}}$ at ${V}_{g}=-5.2 {\mathrm{V}}$. Theoretical analysis shows that this large THE is attributed to the two-dimensional (2D) Bloch-type spin textures (skyrmions or chiral domain walls) stabilized by the large DMI that comes from the presence of the chiral supercells and strong SOC. Direct evaluation of the anomalous Hall conductivity (AHC) and Berry curvature reveals the origin of the large AHC in experiments. Tailoring DMI in 2H-TaS_2_ by dual-intercalation may reveal the universality of DMI and open up the opportunity of more investigations of chiral spin textures in a large family of TMDs.

## Results

### Characterization of the magnetic properties

**Fig. 1:**
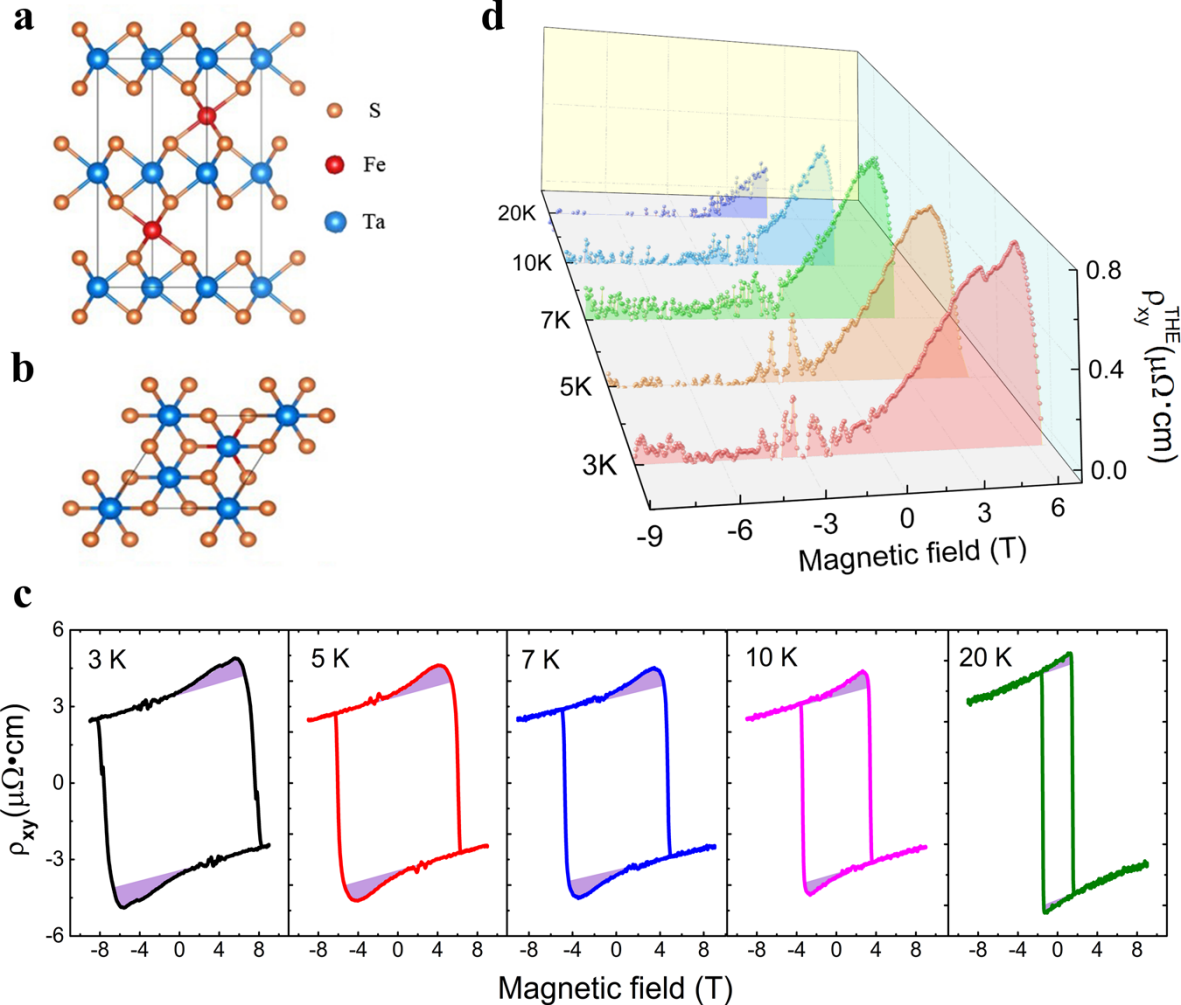
Topological Hall effects observed in Fe-intercalated 2H-TaS_2_. **a**, **b** Crystal structures of Fe_1/3_TaS_2_ of front view (**a**) and top view (**b**). **c** Temperature-dependent Hall resistivity ${\rho }_{{xy}}$ in Fe_0.28_TaS_2_ nanoflakes. Topological Hall resistivity components ${\rho }_{{xy}}^{T}$ are shadowed by the light purple colour. **d** Temperature-dependent Topological Hall resistivity components ${\rho }_{{xy}}^{T}$.

Intercalation of Fe atoms in 2H-TaS_2_ possesses a large perpendicular magnetic anisotropy (PMA)^16,25^. Besides, in Fe_*x*_TaS_2_ with moderate Fe concentrations ($0.28\le x\le 0.33$), Fe atoms intercalate between layers and can form $\sqrt{3}{\rm{a}}\times \sqrt{3}{\rm{a}}$-type supercell ($a$ is the hexagonal lattice parameter of 2H-TaS_2_)^26,27^, as shown in Fig. 1a, b. This resulting chiral $\sqrt{3}{{a}}\times \sqrt{3}{{a}}$-type supercell harbouring strong SOC of Ta and Fe atoms and in the absence of the SIS^21^, allows for a sizable DMI^3–5^. To verify this point explicitly, we first focus on the transport properties in Fe_0.28_TaS_2_. Figure 1c shows the temperature-dependent Hall resistivity in sample S1 with a thickness of $80{{\mathrm{nm}}}$. In conventional FM metals, the Hall resistivity ${\rho }_{{xy}}$ has two components, the normal Hall resistivity ${\rho }_{{xy}}^{N}$ due to Lorentz force induced by an external magnetic field and the anomalous Hall resistivity ${\rho }_{{xy}}^{A}$ scaling with magnetization^28^. This picture is in line with our observations above $20{\mathrm{K}}$, where Hall resistivity exhibits a nearly square-shaped hysteresis loop with a sharp transition near the coercive field ${{\bf{B}}}_{{\bf{c}}}$ (Fig. S2 in SI). While below $20{\mathrm{K}}$, an extra “hump” in ${\rho }_{{xy}}$ near the coercive field emerges, which is not proportional to the magnetization process and is usually attributed to the THE (${\rho }_{{xy}}^{T}$) induced by unconventional spin textures, as shadowed by the light purple in Fig. 1b. The total Hall resistivity now consists of three parts: ${\rho }_{{xy}}={{\rho }_{{xy}}^{N}+\rho }_{{xy}}^{A}+{\rho }_{{xy}}^{T}$. In order to obtain the topological Hall resistivity component, we linearly fitted the Hall resistivity at high field to subtract the normal Hall component ${\rho }_{xy}^{N}$. Due to the large PMA, the anomalous Hall resistivity of Fe_0.28_TaS_2_ exhibits square-shaped hysteresis loops. Thus the anomalous Hall part can be subtracted by linearly extrapolating the high-field anomalous Hall resistivity over the coercive field ${{\bf{B}}}_{{\bf{c}}}$, the obtained hump structures are ascribed to topological Hall resistivity.

Figure 1d shows the extracted ${\rho }_{{xy}}^{T}$ of sample S1 at various temperatures. ${\rho }_{{xy}}^{T}$ decreases with increasing temperatures, and it drops to zero while above $20{\mathrm{K}}$. As discussed above, $\sqrt{3}a\times \sqrt{3}a$-type supercell widely exists in Fe_*x*_TaS_2_ with different Fe doping concentrations ($0.28\le x\le 0.33$). Hence DMI can be developed in Fe_*x*_TaS_2_ in this range of Fe doping level. Beside Fe_0.28_TaS_2_, we carried out extra electric transport measurements in crystals with higher Fe concentrations (Fe_0.3_TaS_2_). As expected, a large THE was also observed in Fe_0.3_TaS_2_ (Fig. S3 in SI). The observation of THE in Fe_*x*_TaS_2_ is a direct transport evidence for DMI in Fe intercalated 2H-TaS_2_.

**Fig. 2:**
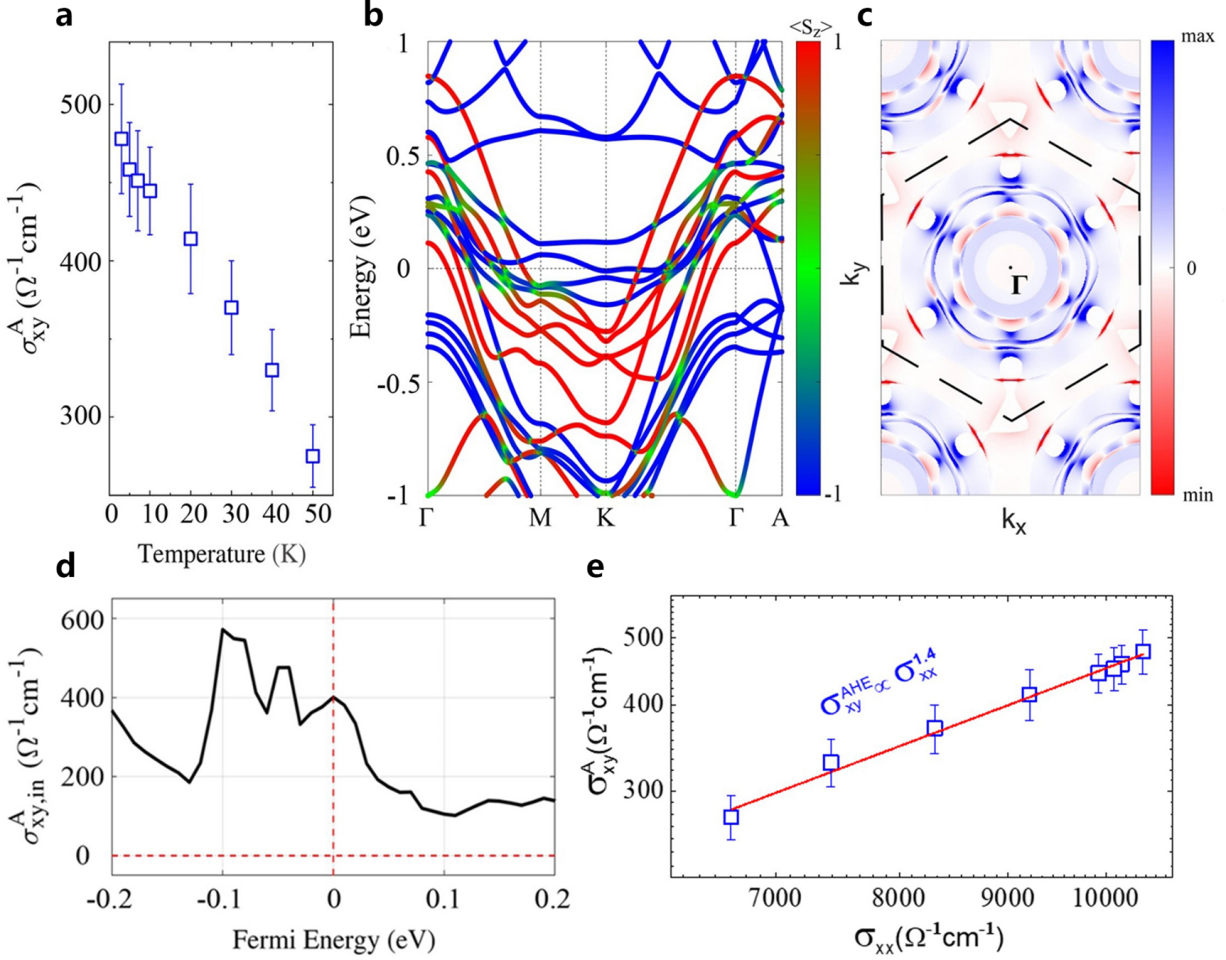
Anomalous Hall conductivity and nontrivial band structure. **a** Temperature-dependent anomalous Hall conductivity in S1. **b**, **c** Band-structure of Fe_1/3_TaS_2_. The colours mark the spin expectation $\left\langle {S}_{z}\right\rangle$ of the band. **d** Berry curvature distribution in ${k}_{z}=0$ plane. **d** Fermi energy ${E}_{{\mathrm{F}}}$ -dependent intrinsic AHC ${\sigma }_{{xy},{in}}^{A}$. **e** Scaling relationship between anomalous Hall conductivities ${\sigma }_{{xy}}^{{{\mathrm{AHE}}}}$ and longitudinal conductivities ${\sigma }_{{xx}}$.

Another electromagnetic response induced by intercalation is large anomalous Hall effect (AHE). The spontaneous FM order of intercalated Fe atoms with strong SOC also acts as the source of AHE^9^. Figure 2a presents temperature-dependent AHC, ${\sigma }_{{xy}}^{A}={\rho }_{{xy}}^{A}/\left({{\rho }_{{xy}}^{A}}^{2}+{\rho }_{{xx}}^{2}\right)$ in sample S1. At $T=2{\mathrm{K}}$, ${\sigma }_{{xy}}^{A}$ reaches $478{\Omega }^{-1}{{{\mathrm{cm}}}}^{-1}$, and it drops to 275${\Omega }^{-1}{{{\mathrm{cm}}}}^{-1}$ at $T=50{\mathrm{K}}$. By using the anomalous Hall angle $\theta ={\sigma }_{{xy}}^{A}/{\sigma }_{{xx}}$ (${\sigma }_{{xx}}$ is the longitudinal conductivity) to measure the contribution of anomalous Hall current with respect to the normal current^9^, we find that the anomalous Hall angle in S1 is as large as $5 \%$ (Fig. S4 in SI). To fully understand the intrinsic AHC induced by the Berry curvatures of electrons in Fe_1/3-δ_TaS_2_ $({\rm{\delta }}\le 0.05)$, we get the band structure of Fe_1/3_TaS_2_ through the First-principles calculations. Due to the spontaneous magnetization of Fe atoms, the spin degeneracy of the band structure is broken, splitting the bands of different spins. The different effective masses of hole pockets with different spins cross each other around the Fermi energy, forming nodal lines in quantity at different **k**_**z**_ planes of the Brillouin zone. The space group P6_3_22 of the Fe_1/3_TaS_2_ allows nonsymmorphic protected spin-polarized Weyl points in the ${\mathbf{\Gamma }}-{\bf{A}}$ direction. Since these Weyl points are far away from the Fermi level, they would not dominate the intrinsic AHC. When the SOC is taken into account, most of the nodal lines will open gaps, contributing AHC through the Berry curvature. Figure 2c displays the distribution of the Berry curvature on the ${{\bf{k}}}_{{\bf{x}}}-{{\bf{k}}}_{{\bf{y}}}$ plane with ${{\bf{k}}}_{{\bf{z}}}=0$ at ${E}_{{\mathrm{F}}}=0.$ Although the gapped nodal lines are completely not in the same energy, the distribution of the Berry curvature forms several circles, which results in a large intrinsic AHC ${\sigma }_{{xy},{in}}^{A}$, as shown in Fig. 2d. The calculated intrinsic AHC ${\sigma }_{{xy},{in}}^{A}$ is about $400{\Omega }^{-1}{{{\mathrm{cm}}}}^{-1}$ at ${E}_{{\mathrm{F}}}=0$, quantitatively comparable with the experimental results. We also consider the scaling relation between AHC and ${\sigma}_{{xx}}$ (${\sigma}_{{xy} }^{A}\propto({{\sigma}_{{xx}}})^{\alpha}$). Plotting ${\sigma}_{{xy}}^{A}$ vs ${\sigma }_{{xx}}$ against temperature in Fig. 2e, we find the scaling exponent ${\alpha }\,\approx\, 1.4$, close to the value of 1.6 for the intrinsic AHC in multiband disordered metals^9^. It is consistent with the complex multiband structure as shown in Fig. 2b.

### Protonic gating

**Fig. 3:**
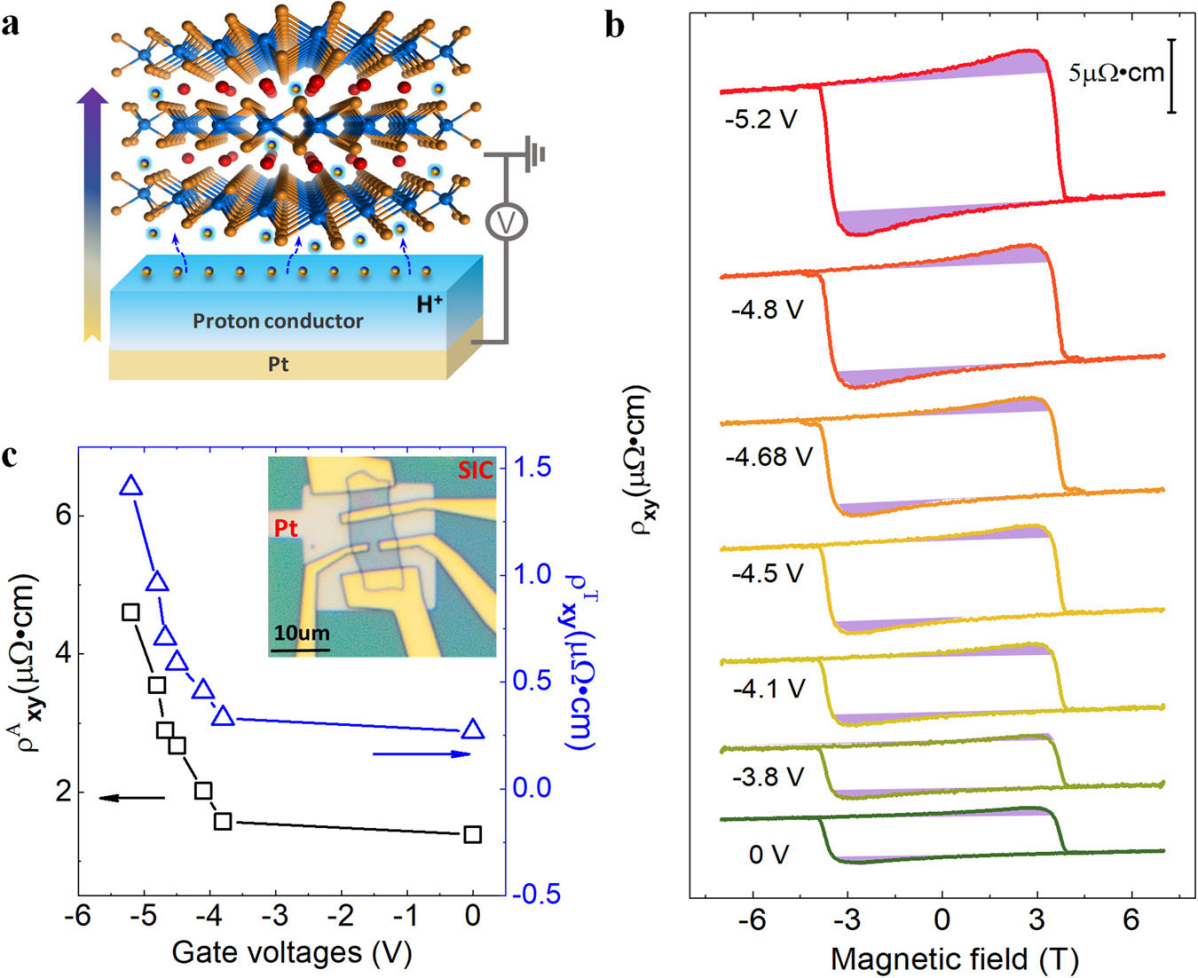
Gate-tuned anomalous Hall and topological Hall effect in Fe_0.28_TaS_2_ nanoflake. **a** A schematic of gate-induced proton intercalation. **b** Hall resistivity under different gate voltages. Topological Hall resistivity ${\rho }_{{xy}}^{T}$ are shadowed by the light purple colour. **c** Gate-dependent anomalous and topological Hall resistivity. Inset: a Hall-bar device on solid ion (proton) conductor (SIC).

Now we focus on controlling of DMI by gate-induced proton intercalation. Compared with widely used Lithium ions, protons are more movable and controllable by gating due to much smaller size, allowing for a large modulation of charge carriers and the magnetic interactions (such as FM and DMI) in bulk itinerate magnets. To achieve this, we developed a new protonic gate (Fig. 3a, see “Methods”), and find that both the observed THE and AHE can be dramatically modulated by gate-controlled proton intercalation, suggesting high tunability of DMI. Figure 3b exhibits the gate-tuned topological Hall and anomalous Hall resistivity ${\rho }_{{xy}}^{T}+{\rho }_{{xy}}^{A}$ in sample S2 at $8{\mathrm{K}}$ with a thickness of $115{{\mathrm{nm}}}$. At ${V}_{g}=0{\mathrm{V}}$, when the magnetic field is swept between $-7{\mathrm{T}}$ and $+7{\mathrm{T}}$, a large THE (as shadowed by the light purple colour) appears around $\pm 3{\mathrm{T}}$. Increasing the voltage from $0{\mathrm{V}}$ to $-5.2{\mathrm{V}}$, we find that both anomalous Hall resistivity and topological Hall resistivity enhanced with increasing gate voltages. Note that, the coercivities keep unchanged during the whole gating process, that is the PMA is almost unchanged in this process, despite both THE and AHE can be dramatically tuned. On the other hand, the stabilization of chiral spin textures is determined by the competition between PMA and DMI, thus the unvaried coercivities indicate that the gate-tuned THE is mainly ascribed to the change of DMI under various gate voltages. Figure 3c shows the gate-dependent amplitudes of the topological and anomalous Hall resistivity. As we can see, the topological Hall resistivity changes from $0.269{\mathrm{\mu}} \Omega \cdot {{\mathrm{cm}}}$ at $0{\mathrm{V}}$ to 1.41 ${\mathrm{\mu}} \Omega \cdot {{\mathrm{cm}}}$ at $-5.2{\mathrm{V}}$. Note that such a huge topological Hall resistivity (${\rho }_{{xy}}^{T}$) at ${V}_{g}=-5.2{\mathrm{V}}$ is larger than most of the known magnetic systems, which is almost the largest one observed in chiral magnets so far (Table 1 in SI). Simultaneously, the anomalous Hall resistivity (${\rho }_{{xy}}^{A}$) monotonically changes from $1.38{\mathrm{\mu}} \Omega \cdot {{\mathrm{cm}}}$ $(0{\mathrm{V}})$ to $4.6{\mathrm{\mu}} \Omega \cdot {{\mathrm{cm}}}$ $(-5.2{\mathrm{V}})$. The variation of the topological Hall resistivity ($\varDelta {\rho }_{{xy}}^{T}={\rho }_{{xy}}^{T}(-5.2{\mathrm{V}})-{\rho }_{{xy}}^{T}$($0{\mathrm{V}}$)) normalized by the zero-bias value is as large as $424 \%$ ($233 \%$ for ${\rho }_{{xy}}^{A}$), which is much larger than the one ($\sim 55 \%$) in oxide heterostructures of SrRuO_3_-SrIrO_3_ tuned by the applied electric field^29^. Note that both anomalous Hall and topological Hall exhibit similar gate-dependence, this is due to their similar dependence on gate-induced carrier density. The evolution of gate-dependent AHE in Fig. 3c (also in Figs. S9 and  S10 in SI) can be fairly well captured by our simulation of AHC in Fig. 2d. The intense modulation of THE qualitatively demonstrates a large and promising electrical tailoring of DMI, which has not been achieved so far. Gate-induced proton intercalation thus may provide a powerful way of electric control of transport phenomena in spintronic and electronic devices with large charge densities.

## Discussion

To further confirm the DMI in intercalation-induced chiral supercells, we consider the impacts of crystal symmetry breaking on the spin textures. First, the large PMA implies an anisotropic ferromagnet, leading to merely two-fold degenerate ground states (Ising-type state). Second, Fe_1/3_TaS_2_ has a layered hexagonal structure of 2H-type TaS_2_ intercalated by Fe atoms which belongs to the noncentrosymmetric chiral space group P6_3_22^21^. That is, the Fe atoms intercalating in $\sqrt{3}{{a}}\times \sqrt{3}{{a}}$-type supercell will break the SIS. As a result, a substantial DMI is allowed due to the combination of strong SOC of Fe and Ta atoms and the broken SIS^3–5^. This DMI may share the same RKKY mechanism as the out-of-plane FM ordering demonstrated in Fe_1/4_TaS_2_^22^. However, a substantial DMI is allowed in Fe_1/3_TaS_2_ due to the combination of strong SOC of Fe and Ta atoms and the broken SIS^3–5^, but absent in Fe_1/4_TaS_2_ due to the presence of SIS. Thus, the specific magnetic structure can be effectively described by a spin model,

1 $${\mathbf{H}}=-J{\sum }_{ < i,j > }{{\mathbf{S}}}_{{\mathbf{i}}}\cdot {{\mathbf{S}}}_{{\mathbf{j}}}+\mathop{\sum }_{i,j}{{\mathbf{d}}}_{{\mathbf{ij}}}{{\mathbf{S}}}_{{\mathbf{i}}}\times {{\mathbf{S}}}_{{\mathbf{j}}}+K\mathop{\sum }_{i}{{\mathbf{S}}}_{{\mathbf{i}}}^{{\mathbf{z}}}\cdot {{\mathbf{S}}}_{{\mathbf{i}}}^{{\mathbf{z}}}-{{\mathbf{B}}}_{{\bf{z}}}{\sum }_{i}{{\mathbf{S}}}_{{\mathbf{i}}}^{{\mathbf{z}}}$$

where the indices *i* and *j* sum over the Fe atoms. $J \;> \; 0$ is the FM exchange interaction, ${{\bf{d}}}_{{\bf{ij}}}$ is the vector of DMI, $K \;<\; 0$ indicates PMA favouring an easy-axis, and the last term is the Zeeman energy due to the applied magnetic fields.

In terms of symmetry (point group ${D}_{6}$), the DMI are allowed ${{\bf{d}}}_{{\boldsymbol{\perp }}}$ and ${{\bf{d}}}_{{\boldsymbol{/}}{\boldsymbol{/}}}$, for the components perpendicular and parallel to the direction of the **c** axis^30^. The total DMI ${{\bf{d}}}_{{\bf{tot}}}$ now can be written as ${{\bf{d}}}_{{\bf{tot}}}={{c}_{1}{\bf{d}}}_{{\boldsymbol{\perp }}}+{c}_{2}{{\bf{d}}}_{{\boldsymbol{/}}{\boldsymbol{/}}}={c}_{1}({{\bf{m}}}_{{\bf{z}}}\frac{\partial {{\bf{m}}}_{{\bf{x}}}}{\partial y}-{{\bf{m}}}_{{\bf{x}}}\frac{\partial {{\bf{m}}}_{{\bf{z}}}}{\partial y}-{{\bf{m}}}_{{\bf{z}}}\frac{\partial {{\bf{m}}}_{{\bf{y}}}}{\partial x}+{{\bf{m}}}_{{\bf{y}}}\frac{\partial {{\bf{m}}}_{{\bf{z}}}}{\partial x})+{c}_{2}({{\bf{m}}}_{{\bf{x}}}\frac{\partial {{\bf{m}}}_{{\bf{y}}}}{\partial z}-{{\bf{m}}}_{{\bf{y}}}\frac{\partial {{\bf{m}}}_{{\bf{x}}}}{\partial z})$, with arbitrary coefficients ${c}_{\mathrm{1,2}}$ and reduced magnetization ${{\bf{m}}}_{{\bf{x}}{\boldsymbol{,}}{\bf{y}}{\boldsymbol{,}}{\bf{z}}}$.

**Fig. 4:**
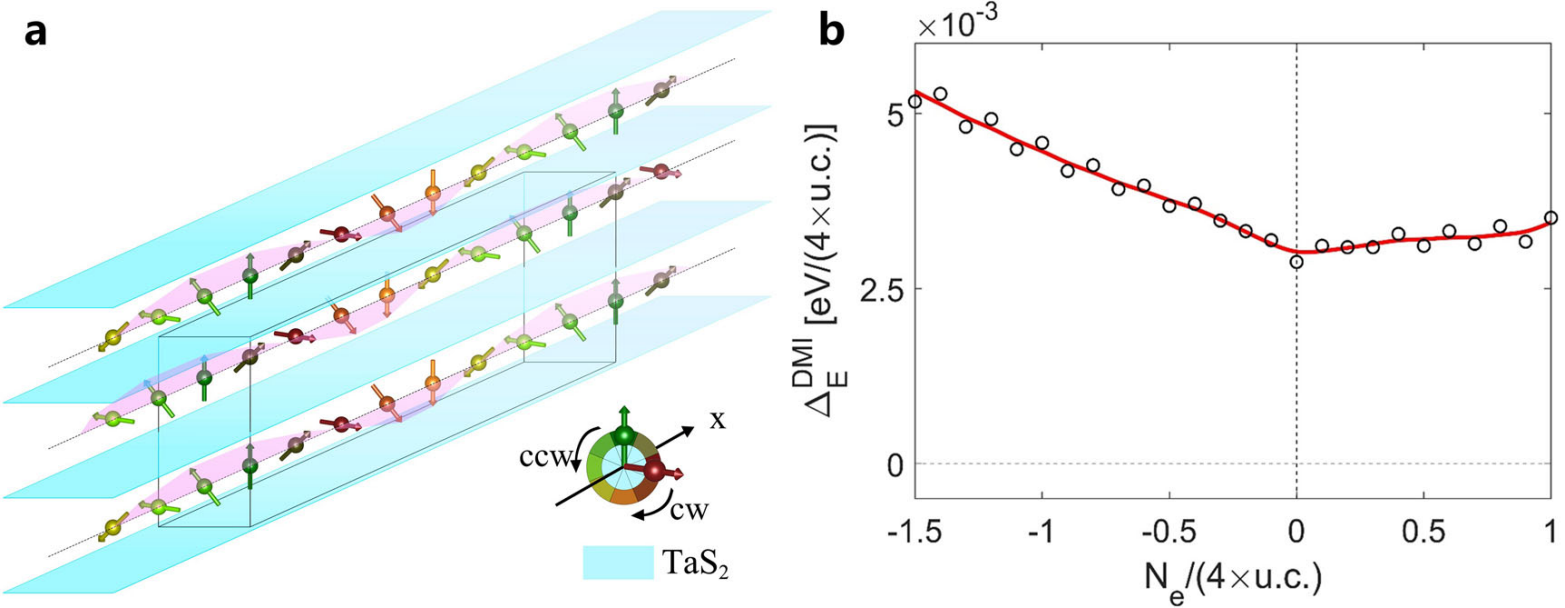
DMI simulation in First-principles calculations. **a** Spin configurations used to calculate DMI strength. Spins are represented by arrows. **b** The electron number ${N}_{e}$ -dependent DMI strength ${{\bf{d}}}_{{\boldsymbol{\perp }}}$ ($\propto {\triangle }_{E}^{{{\mathrm{DMI}}}}$) for the supercell with four-spin cycle along one selected direction in (**a**). ${N}_{e}=0$ represents the case of ${E}_{{\mathrm{F}}}=0$, and ${N}_{e}=-1.5(+1)$ represents the Fermi energy around $-80{{\mathrm{meV}}}(+50{{\mathrm{meV}}})$. The black circles are the results of First-principles calculation and the red line is fitted from the black circles for guiding eyes.

It is known that the large PMA would suppress the chiral conical order^31^ or the chiral soliton phase^19^, in which ${{\bf{d}}}_{{\boldsymbol{/}}{\boldsymbol{/}}}$ twists the in-plane spin magnetic moments. In the work, we shall closely examine the DMI ${{\bf{d}}}_{{\boldsymbol{\perp }}}$. We carry out the First-principles calculation of the DMI ${{\bf{d}}}_{{\boldsymbol{\perp }}}$($\propto {\triangle }_{E}^{{{\mathrm{DMI}}}}$) by evaluating the total energy differences between the clockwise and counter-clockwise Bloch-type spin textures along the lines of Fe atoms as shown in Fig. 4a^32^. The emergent THE at lower temperatures suggests that the DMI is sufficient to destabilize the FM state, forming the Bloch-type spin textures in bulk (N${\acute {{\rm{e}}} }$el-type spin textures are probably ruled out, as discussed in Fig. S12 in SI), such as skyrmions and chiral domain walls^33^. Due to the weak coupling among the TaS_2_ layers, each layer of Fe atoms is an effective 2D magnetic system. Then, we first consider the 2D Bloch-type skyrmions. We further simulate the impact of gating by changing the electron number ${N}_{e}$ in the First-principles calculations. As shown in Fig. 4b, when the gate voltage increases (shifts the Fermi energy towards the negative values), the strength of DMI becomes larger. Besides the gate-induced change of carrier density, the proton intercalation may locally form proton concentration gradient which can also lead to the SIS breaking and contribute DMI^34^. Specifically, the DMI at ${E}_{{\mathrm{F}}}=-80{{\mathrm{meV}}}$ is about twice of the value at ${E}_{{\mathrm{F}}}=-0{{\mathrm{eV}}}$. The topological Hall resistivity from 2D skyrmions is proportional to the strength of the emergent magnetic field ${{\bf{b}}}_{{\bf{z}}}$, where ${{\bf{b}}}_{{\bf{z}}}={n}_{{\mathrm{s}}}{\varnothing}_{0}/2\pi {R}^{2}$, with *R* being the diameter of skyrmion, ${n}_{{\mathrm{s}}}$ being the density of skyrmions and ${{{\varnothing }}}_{0}={hc}/e$ being the flux quantization of emergent magnetic field of unit sphere for each skyrmion. Thus, with the increasing strength of DMI, the density of 2D skyrmions increases greatly than linearly, leading to dramatic increase of THE. This increased density of skyrmion could facilitate the promising next-generation low-energy and high-density storage spintronic devices based on skyrmion systems and the manipulation of dynamics of skyrmions through gate-tunable DMI. Recently, the chiral domain wall with ${Z}_{6}$ vortex was suggested to account for the observation of THE in Mn_3_Sn^35,36^. Analogically, the chiral domain wall with ${Z}_{6}({Z}_{2}\times {Z}_{3})$ vortex in Fe_1/3_TaS_2_ revealed by transmission electron microscopy^21^ may also be a possible origin of large THE.

In conclusion, intercalating Fe atoms in a TMD 2H-TaS_2_, Fe_1/3-δ_TaS_2_ (${\rm{\delta }}\le 0.05$) nanoplates exhibit large THE, demonstrating a strong transport evidence of sizable DMI and the emergence of Bloch-type spin textures (such as skyrmions and chiral domain walls). Gate-induced proton intercalation can largely modulate the amplitudes of topological Hall resistivities by approximately $420 \%$, indicating the high tunability of DMI. Theoretical analysis and First-principles calculations suggest that the sizable DMI dominantly comes from the intercalated Fe atoms, playing a key role in magnetic orders and the formation of the chiral supercells with broken SIS. Our discovery demonstrates that dual-intercalation (intercalation of magnetic atoms and protons) is a promising way of tailoring DMI and manipulating chiral spin textures in 2H-TaS_2_, greatly inspiring further investigations in a large family of TMDs.

## Methods

### Single crystal growth

Single crystals of Fe_*x*_TaS_2_ were grown via chemical vapour transport method with iodine as the transport agent with suitable mole ratio and sealed in an evacuated quartz tube (Supplementary Section 1).

### Device fabrication and transport measurements

Solid protonic electrolyte was prepared by the sol-gel processes by mixing tetraethyl orthosilicate (from Alfa Aesar), ethanol, deionized water, phosphoric acid (as a proton source, from Alfa Aesar, $85 \%$ ${\rm{wt}} \%$). The mixed solution was stirred and annealed before use (Supplementary Section 2). Transport measurements were performed in a commercial Physical Property Measurement System (PPMS) with magnetic field up to $9{\mathrm{T}}$. Protonic gating experiments were performed in commercial magnetic property measurement system (MPMS) with a maximal magnetic field of $7{\mathrm{T}}$. To decrease the leaking current during the gating, voltage was swept at $250{\mathrm{K}}$. Once the resistance was changed, the sample was quickly cooled down to low temperatures for magneto-transport measurements.

## Supplementary information

Supplementary Information

## Data Availability

All data supporting the findings of this study are available from the corresponding author on request.
