# Supplementary material for: Tailoring Dzyaloshinskii–Moriya interaction in a transition metal dichalcogenide by dual-intercalation
Source: Nat Commun. 2021 Jun 15;12:3639. doi: 10.1038/s41467-021-23658-z (PMC8206329; doi:10.1038/s41467-021-23658-z)
Supplement: Supplementary file 1 — Supplementary Information [file 41467_2021_23658_MOESM1_ESM.pdf]

Supplementary Information

Tailoring Dzyaloshinskii-Moriya interaction in a transition metal dichalcogenide by dual-intercalation

Guolin Zheng[#], Maoyuan Wang[#], Xiangde Zhu[#], Cheng Tan, Jie Wang, Sultan Albarakati, Nuriyah Aloufi, Meri Algarni, Lawrence Farrar, Min Wu, Yugui Yao, Mingliang Tian[*], Jianhui Zhou[*], Lan Wang[*]

# Those authors equally contribute to the paper.

* Corresponding authors. Correspondence and requests for materials should be addressed to J. Z. (email: jhzhou@hmfl.ac.cn); M. T. (email: tianml@hmfl.ac.cn); L. W. (email: lan.wang@rmit.edu.au).

1. Single Crystal Growth

Single crystals of $Fe_{1/3-\delta}TaS_2$ were grown via chemical vapor transport method with iodine as the transport agent. First, iron granular, Ta powder and sulfur sponges with mole ratio of 0.35:1:2 were mixed and sealed in an evacuated quartz tube. The tube was heated at $1173\ K$ for one week. Second, the obtained powder together with about $120\ mg$ iodine were sealed in an evacuated quartz tube with $15\ mm$ inner diameter and $18\ cm$ length. Then the tube was placed in a horizontal two-zone furnace, with source zone kept at $1223\ K$ and growth zone kept at $1123\ K$ for 10 days. The obtained as grown crystals were cleaned in ethanol with ultrasonic.

2. Device Fabrication and Transport Measurements

Solid protonic electrolyte was prepared by the sol-gel processes [1-3]. We first mixed tetraethyl orthosilicate (from Alfa Aesar), ethanol, deionized water, phosphoric acid (as a proton source, from Alfa Aesar, 85% wt%) with a typical molar ratio 1:18:6:0.03, then the mixed solution was stirred for 2 hours and annealed for another 2 hours at 50 ℃ in a sealed bottle to form polymerized Si–O–Si chains. Finally, the substrate with bottom gate electrodes was spin-coated with the prepared protonic solution and baked at 150 ℃ for 25 mins.

$Fe_{1/3-\delta}TaS_2$ nanoplates were mechanically exfoliated from bulk crystals and placed on a silicon substrate with a $300\ nm$ SiO_2 layer. Then the $Fe_{0.33-\delta}TaS_2$ nanoplates with suitable size and thickness were dry transferred by polydimethylsiloxane (PDMS) onto the protonic electrolyte (with thickness $300\ nm$). Both the exfoliation and transfer processes were carried out in a glove box full of high purity Argon gas with $O_2 < 0.1\ ppm$, $H_2O < 0.1\ ppm$. Hall-bar structures were fabricated by standard Electron-beam lithography (EBL) method followed by Cr/Au

($10\ nm/100\ nm$) evaporation in a high vacuum sputtering system with base pressure less than $5 \times 10^{-8}\ Torr$.

3. Theoretical Calculations

First-principles calculations are performed by using Vienna ab initio simulation package (VASP) [4, 5] based on the density function theory with Perdew-Burke-Ernzerhof (PBE) parametrization of generalized gradient approximation (GGA) [6]. The energy cutoff of the plane wave basis is set as $350\ eV$, and the Brillouin zone of $Fe_{1/3}TaS_2$ is sampled by $12 \times 12 \times 6$ K mesh. The ionic positions are fully optimized until the force on each atom was less than $0.01\ eV/Å$ while the lattice parameters are fixed as the experimental value. The intrinsic anomalous Hall conductivity [7] is calculated based on the Wannier90 code [8, 9] with s, d orbitals of Fe and Ta atoms and s and p orbitals of S atoms. The k mesh for intrinsic hall conductivity calculation is $100 \times 100 \times 50$ with an adaptive refinement mesh of size of $5 \times 5 \times 5$. Note that in the simulation of the impact of gating, we adopt the rigid band approximation and neglect the modification of band structures. The total energy calculation for DMI is based on GGA+U method [10, 11] with U values of $2.5\ eV$ and $4\ eV$ for d orbitals of Ta and Fe atoms. Note that in the simulation of the impact of gating, we adopt the rigid band approximation and neglect the modification of band structures.

4. Temperature dependent out-of-plane magnetization and longitudinal resistance.

Fig. S1 shows the temperature dependence of out-of-plane magnetization and longitudinal resistance of $Fe_{0.28}TaS_2$ single crystal. With a perpendicular magnetic field of $500\ Oe$, the out-of-plane magnetization shows a dramatic upturn around $85\ K$, indicating the Curie temperature $T_c = 85\ K$, as shown in Fig. S1(a). The temperature-dependent longitudinal resistance of $Fe_{0.28}TaS_2$

nanoflake also exhibits a resistance anomaly around 85 K, in line with the magnetization of single crystals. Both transport properties and out-of-plane magnetization are line with previous studies [12-14].

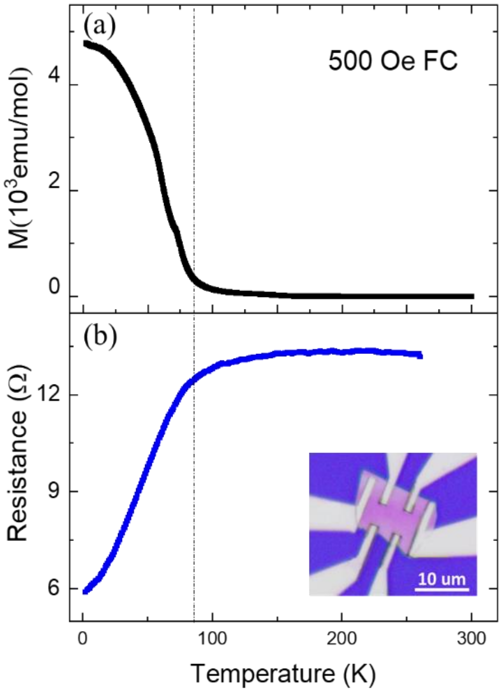

Figure S1. Temperature dependent magnetization and longitudinal resistance. (a) Temperature dependence of out-of-plane magnetization of $Fe_{0.28}TaS_2$ single crystal. (b) Shows the temperature dependence of longitudinal resistance in exfoliated nanoflake. Inset shows the optical image of a typical Hall-bar device, with a thickness of 90 nm.

5. Square-shaped hysteresis loops above 20 K in sample S1.

Due to the strong perpendicular magnetic anisotropy, the anomalous Hall resistivities exhibit square-shaped hysteresis loops. Below 20 K, the anomalous Hall almost keeps unchanged (Fig. 1(c) in main text), while for temperatures above 20 K, it increases as the increase of the temperature, this increment is mainly due to the enhancement of extrinsic scattering induced by

thermal fluctuation. However, the "hump" features on Hall trance at low temperatures shows an opposite tendency with respect to the temperature, revealing their different origins.

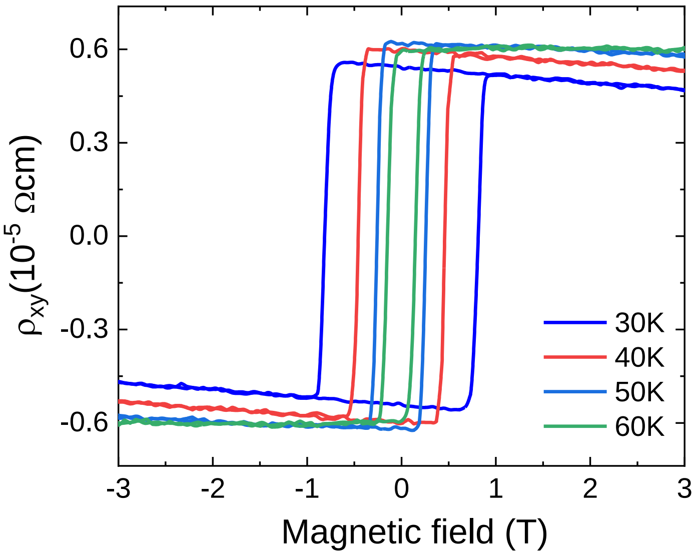

Figure S2. Anomalous Hall effect (AHE) above 20 K in sample S1. The hysteresis loops in sample S1 at selected temperatures (above 20 K) exhibit square-shaped loops with sharp transition near the coercive field.

6. Topological Hall effect (THE) observed in $Fe_{0.3}TaS_2$ single crystals.

As mentioned in main text, $\sqrt{3}a \times \sqrt{3}a$ type superlattice supports a sizable DMI and widely exists in Fe_xTaS_2 with different Fe doping concentrations. To verify this point, we further carried out the electric transport measurements in $Fe_{0.3}TaS_2$ crystals. Fig. S3 shows the transport properties of $Fe_{0.3}TaS_2$ single crystals at low temperature region. Akin to $Fe_{0.28}TaS_2$, the Hall resistivity exhibits extra humps (shadowed by light yellow), which are not proportional to the magnetization and should be attributed to THE due to sizable DMI. Note that the THE observed in $Fe_{0.3}TaS_2$ with higher Fe concentrations emerges at low temperatures (within 4 K), compared with the THE in $Fe_{0.28}TaS_2$.

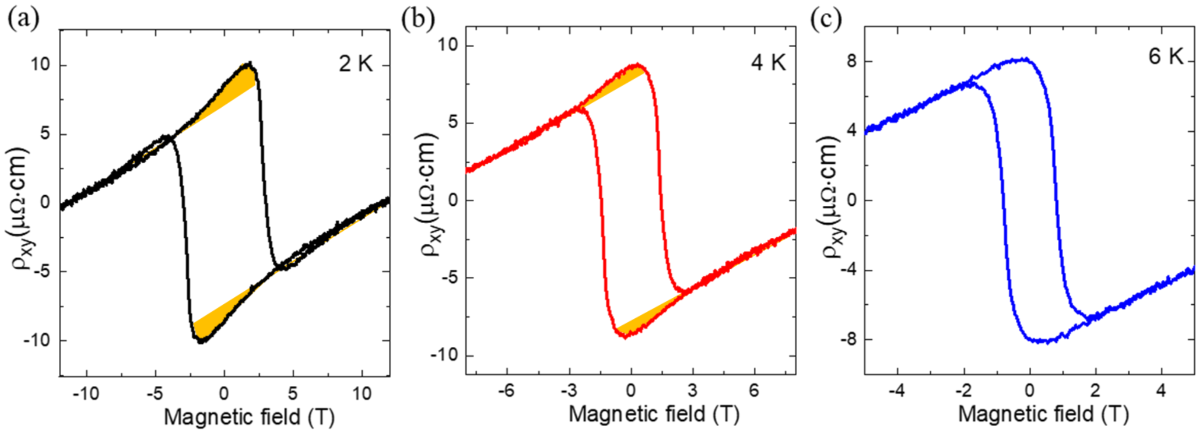

Figure S3. THE observed in $Fe_{0.3}TaS_2$ single crystals. The extra humps shadowed by light yellow are attributed to THE. The THE signal disappears while the temperature exceeds 6 K.

7. Longitudinal resistivity and anomalous Hall angle in $Fe_{0.28}TaS_2$ nanoflake.

Fig. S4(a) exhibits temperature-dependent longitudinal resistivity in sample S1. The corresponding anomalous Hall resistivity and longitudinal magnetoresistivity at 3 K are shown in Fig. S4(b). The calculated anomalous Hall angle $\theta = \sigma_{xy}^A/\sigma_{xx} \sim 5\%$ ($\sigma_{xx} = \rho_{xx}/(\rho_{xx}^2 + \rho_{xy}^2)$, $\sigma_{xy}^A = \rho_{xy}^A/(\rho_{xx}^2 + {\rho_{xy}^A}^2)$). We also repeat the measurements in several samples and find that the anomalous Hall angle in $Fe_{0.28}TaS_2$ is $4.4\% \leq \theta \leq 5\%$, which is almost the same order as recently reported ferromagnetic semimetal Fe_3GeTe_2 [15]. Our calculated intrinsic AH conductivity is about 400 $(\Omega cm)^{-1}$ as shown in Figure 2 in the main text, this value is well consistent with experimental result of $478(\Omega cm)^{-1}$, implying that the AH conductivity is dominated by the intrinsic Berry-phase contribution.

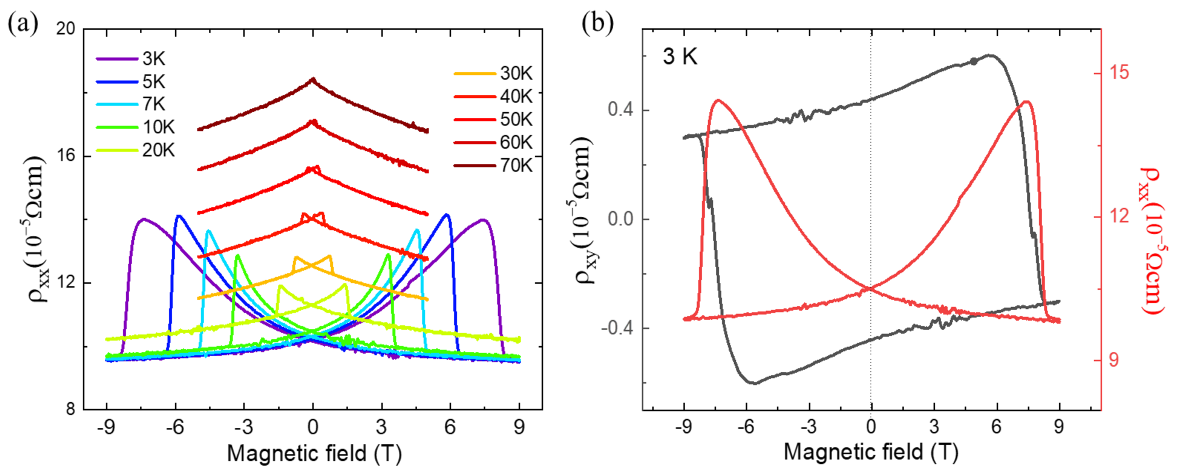

Figure S4. Large anomalous Hall angle in $Fe_{0.28}TaS_2$. (a) Temperature-dependent longitudinal magnetoresistivity. (b) Shows both Hall resistivity and longitudinal magnetoresistivity at 3 K. After subtracting the normal Hall resistivity ρ_{xy}^{N}, the anomalous Hall resistivity is given as $\rho_{xy}^{A} = \rho_{xy}(\mathbf{B} = 0\,T)$.

8. The origin of extra "hump" structures on anomalous Hall loops.

It is possible that the extra "hump" structures are merely due to the admixing of longitudinal resistance. Experimentally, due to the misalignment of Hall-bar, the measured Hall resistivity would pick up a small longitudinal resistivity δR_{xx}. So all the anomalous Hall loops were antisymmetrized in order to remove the longitudinal resistivity component. However, the antisymmetric "humps" structures can still be identified in raw Hall data, as shown in Figure S5. Figure S5(a) shows a slender $Fe_{0.28}TaS_2$ nanoflake with a Hall-bar configuration. Though the Hall-bar device is visually symmetric, it is still inevitable to pick up a small longitudinal resistivity component due to a very small misalignment, as shown in Figure S5(b). The measured Hall resistivity is R_{13}, which contains a small longitudinal component R_{23}. Figure S5(c) shows the raw

Hall resistivity data (black) as well as an antisymmetrized data (red). As we can see, hump structures can still be identified near coercive fields in raw data. Figure S5(d) shows the small longitudinal component R_{23} deduced from Figure S5(c). Stark contrast to the antisymmetric hump structures, R_{23} is symmetric with respect to the field. So the admixing of longitudinal resistance cannot lead to this antisymmetric hump structures.

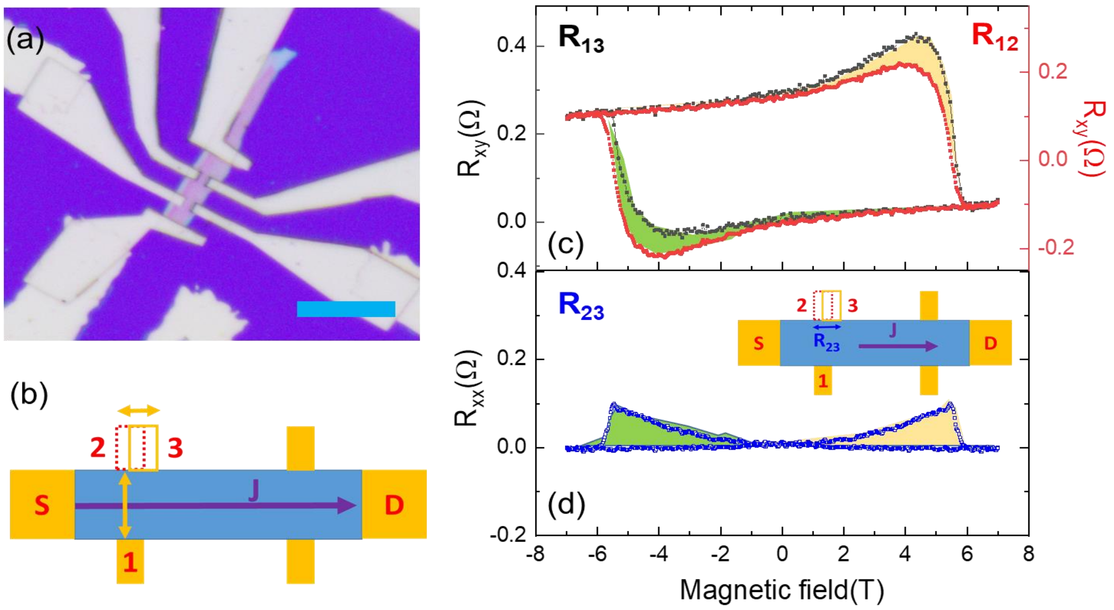

Figure S5. **Misalignment of Hall-bar**. (a) Optical image of our Hall-bar device (Scale bar: 10 μm). (b) A schematic of a Hall-bar device with a tiny misalignment (heavily exaggerated). (c) Measured Hall resistance (R_{13}) and antisymmetrized data (R_{12}). (d) A small longitudinal resistance (R_{23}) deduced from (b).

Another possible reason for the "hump" structures in anomalous Hall loops might be due to the admixing of two anomalous Hall components with opposite signs without involving in chiral spin textures [16]. For example, in a magnetically doped topological insulator thin film, the two anomalous Hall components may separately come from the surface and bulk state [17], which can

lead to "hump" structures on anomalous Hall loops. In Fe-doped TaS_2, it is possible that the intercalations of Fe atoms in TaS_2 might be inhomogeneous, which would potentially lead to two magnetic phases. The topological Hall features below $20\ K$ might merely originate from the combination of two opposite AHE, as illustrated previously [16]. We note that, however, varying the Fe doping concentration in a large range (e.g. $0.25 < x < 0.3$), the AHE in Fe_xTaS_2 nanoflakes doesn't exhibit any sign reversal. Besides, the Curie temperatures of Fe_xTaS_2 with different doping concentrations are much higher than $20\ K$. So it is impossible to form a magnetic layer with an opposite anomalous Hall effect and a much lower Curie temperature of $20\ K$ by inhomogeneous intercalation.

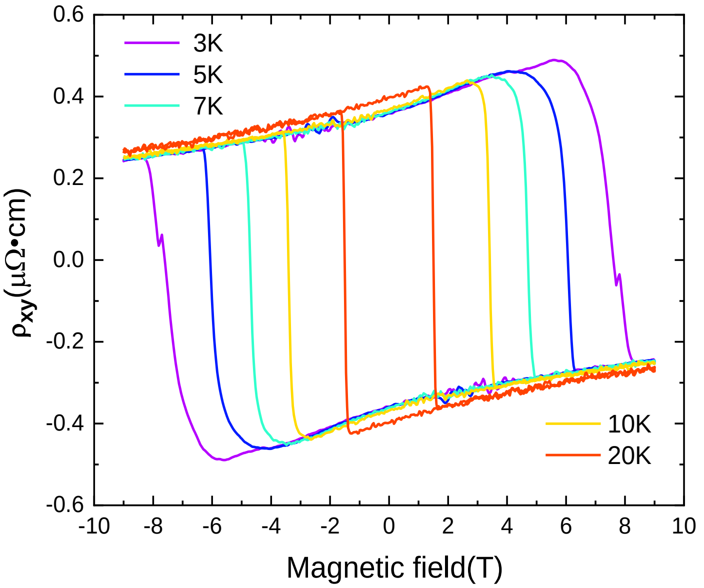

Figure S6. Temperature dependent anomalous Hall resistivity. The anomalous Hall resistivity keeps almost unchanged for temperature below $20\ K$.

Actually, in an interfacial system with two opposite AHE, due to the competition of two AHE, both "hump" features and the combined anomalous Hall resistivity usually exhibit a strong

temperature dependence. However, the AHE in Fe-doped TaS_2 keeps unchanged below $20\ K$, while the "hump" features show a strong temperature dependence, as we can see in Figure S6. The unchanged AHE below $20\ K$ is contradictory to the above scenario.

According to the analysis in the main text, topological Hall effect originates from the chiral spin textures which are stabilized by DMI. If we change the doping concentration of Fe atoms to around $x = 0.25$, the intercalation of Fe atoms in TaS_2 would not form chiral supercells and accordingly, THE should disappear. To testify this assumption, we tested $Fe_{0.26}TaS_2$ nanoflakes, in which $\sqrt{3}a \times \sqrt{3}a$-type (a is the hexagonal lattice parameter of 2H-TaS_2) chiral supercells are very limited and the system is dominated by $2a \times 2a$-type superstructures without a sizable DMI. As shown in Figure S7, the hysteresis loops in $Fe_{0.26}TaS_2$ are almost square-shaped with no clear "hump" features near coercivities. The disappearance of THE in $Fe_{0.26}TaS_2$ further validates the fact that the observed THE is indeed originated from the chiral spin textures.

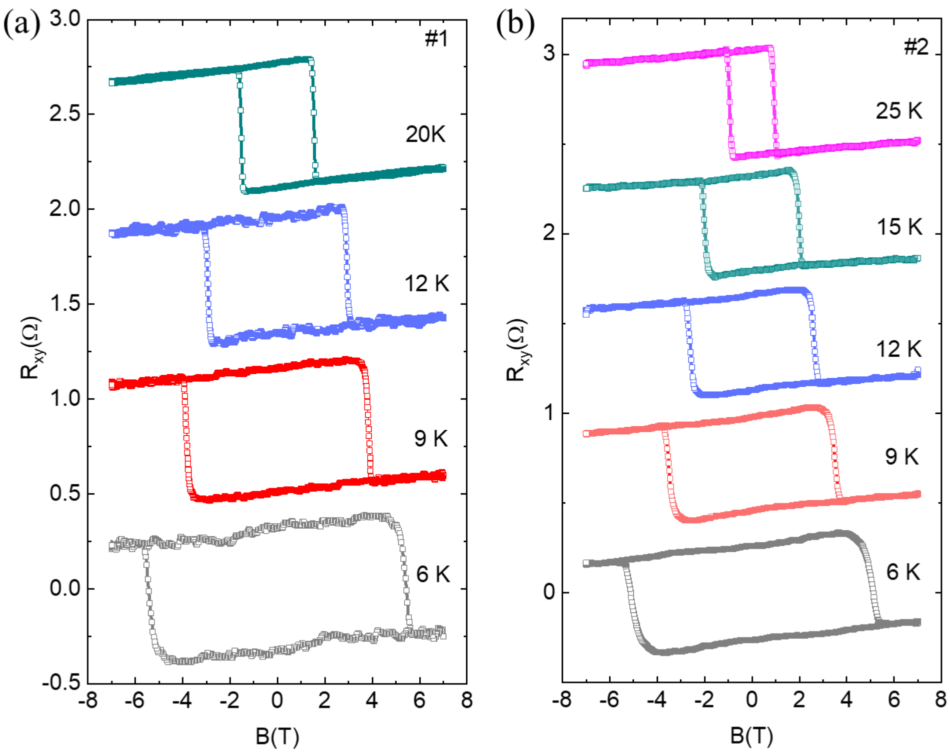

Figure S7. Anomalous Hall effect in $Fe_{0.26}TaS_2$ nanoflakes. (a) and (b) Due to the absence of chiral supercell, topological Hall effects have disappeared.

9. Gate-dependent resistance at $250\ K$

Due to their small size, protons are movable at high temperature region. The high mobility of protons at high temperatures would easily damage our devices. In order to decrease the mobility of protons and finely tune the resistance, we set the temperature at $250\ K$ to apply the gate voltages. Once the resistance was changed, we fixed the gating voltage and decreased the temperature to $2\ K$ for transport measurements. Below $200\ K$, we found the leaking current was decreased to below $0.1\ nA$, indicating that the protons can be stabilized at temperature below $200\ K$. Figure S8 shows the evolution of resistance during the gating process.

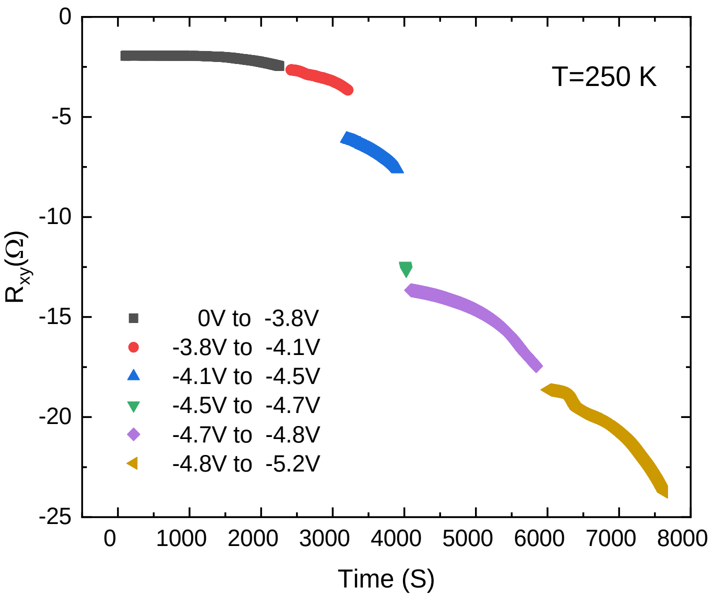

Figure S8. The evolution of resistance at $250\ K$ during the gating process. Sweeping the voltage from $0\ V$ to $-5.2\ V$, the resistance was dramatically changed.

10. Gate-dependent longitudinal resistivity and carrier density in sample S2 at 8 K.

Fig. S9(a) shows gate-dependent longitudinal resistivity in sample S2. Akin to gate-dependent anomalous Hall resistivities shown in Fig. 3b, the longitudinal resistivity can be dramatically enhanced when the gate voltages are swept from $V_g = 0\ V$ to $V_g = -5.2\ V$. Though the band structure is complex, the Hall resistivities between 4.5 T and 7 T are nearly linear with respect to magnetic fields. Analysing the Hall resistivity above coercivity field (as shown in Fig. S9(b)), we further found that the hole-type carrier density changed from $\mathrm{p} = 9.05 \times 10^{22} cm^{-3}$ ($V_g = 0\ V$) to $\mathrm{p} = 5.83 \times 10^{21} cm^{-3}$ ($V_g = -5.2\ V$). Such a dramatic modulation of carrier density in metallic magnet $Fe_{0.28}TaS_2$ reveals the ultra-high efficiency of protonic gate.

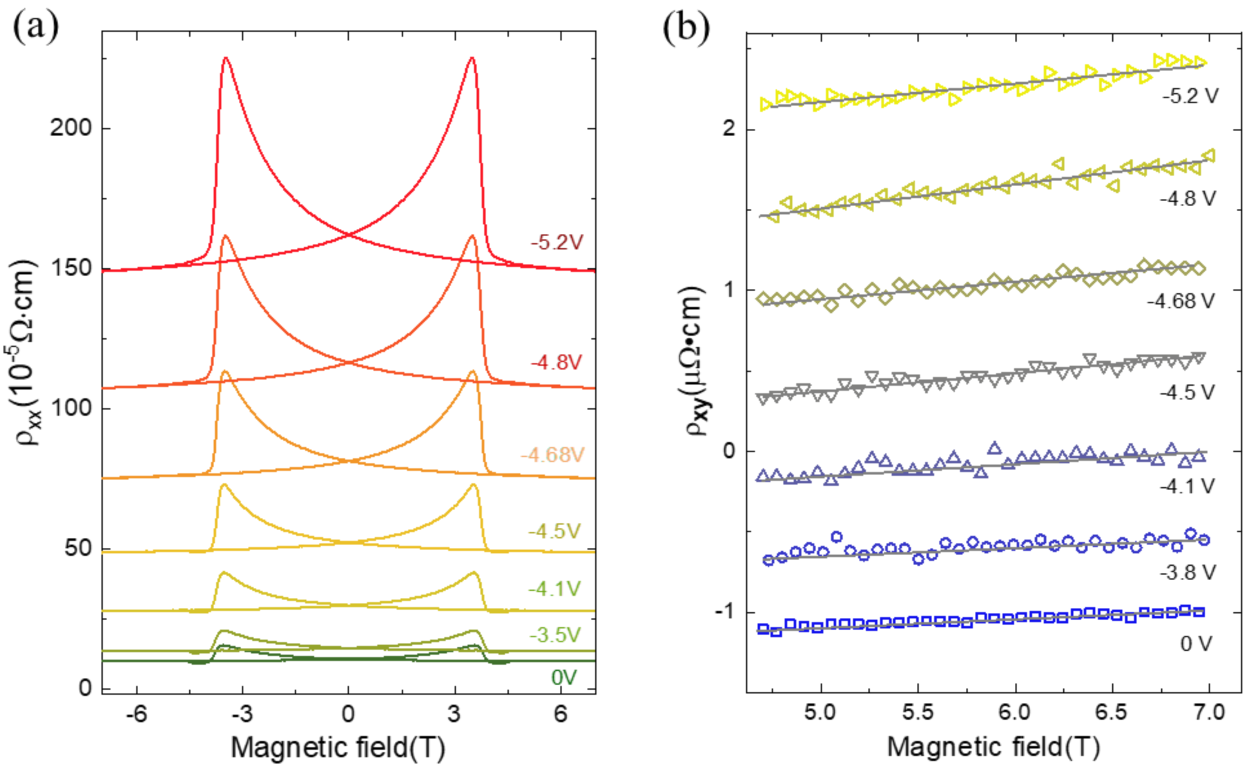

Figure S9. Gate-dependent longitudinal resistivity and carrier density in sample S2. (a) Gate-dependent longitudinal magnetoresistivity. (b) High field Hall resistivities under different gate voltages. Grey lines are linear fitting lines to experimental data under various gate voltages.

11. Density of states (DOS) of $Fe_{1/3}TaS_2$ near $E_F = 0\ eV$.

Figure S10 shows the calculated DOS of $Fe_{1/3}TaS_2$ near Fermi energy $E_F = 0\ eV$. The DOS around $E_F = 0\ eV$ decreases when the E_F is shifted towards negative value. In the other hand, the AHC calculated in Fig. 2d declines while DMI in Fig. 4c increases accordingly. The gate-tuned both AHE and THE in Fig. 3b are well in line with our theoretical calculations. As the sweeping the gate voltages from $0\ V$ to $-5.2\ V$, the hole carrier density decreases gradually (Fig. S9(b)), while the amplitudes of both anomalous Hall resistivity and topological Hall resistivity are elevated (AHC is decreased).

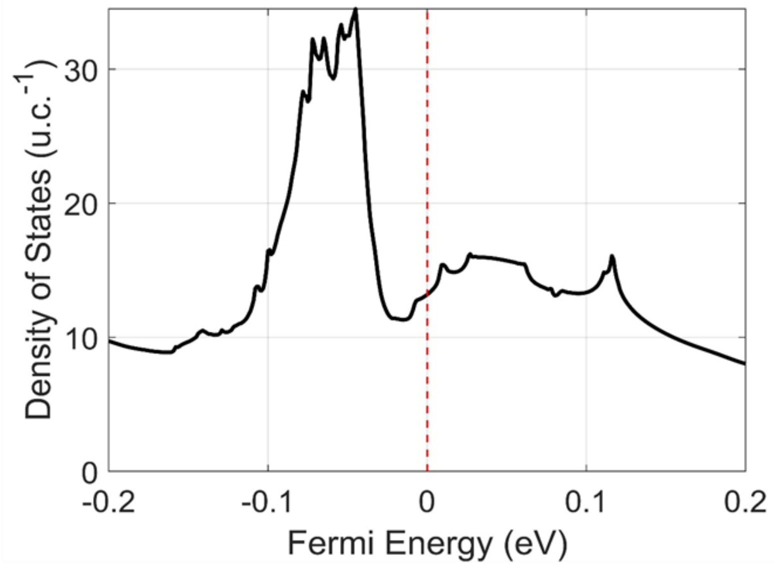

Figure. S10. Density of states (DOS) of $Fe_{1/3}TaS_2$ near $E_F = 0\ eV$. The DOS declines when E_F is shifted towards negative values.

12. Electron diffraction measurements

To characterize the $\sqrt{3}a \times \sqrt{3}a$ type superlattices of Fe_xTaS_2 with $0.28 \leq x \leq 0.33$, we take $Fe_{0.3}TaS_2$ single crystals as an example to carry out electron diffraction studies. Fig. S11 shows the selected area electron diffraction (SEAD) pattern. Besides the main structures (large bright spots, TaS_2 phase), we can see some superlattice reflections (faint, marked by red circle). This $\sqrt{3}a \times \sqrt{3}a$ type superlattices break the spatial inversion symmetry and support a sizable DMI, resulting in a large THE.

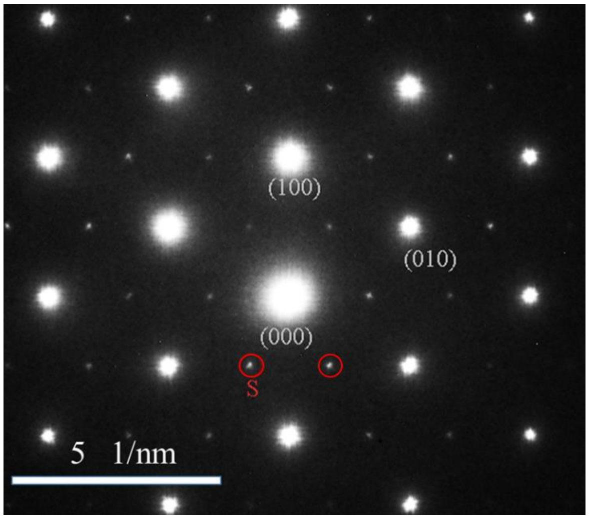

Figure S11. Electron diffraction pattern of $Fe_{0.3}TaS_2$. The reflections from superlattices are marked by the red circles.

13. Comparison of DMI between Bloch-type and Néel-type spin structures.

For a better understanding of the chiral spin structures in gate-tuned Fe_xTaS_2 nanoflakes, we also calculated the DMI in Néel-type spin structures. Fig. S12 shows the electron number N_e dependent DMI strength $\mathbf{d}_\perp(\propto \Delta_E^{DMI})$ for both Bloch-type and Néel-type spin structures. $N_e =$

0 represents the case of $E_F = 0$, and $N_e = -1.5(+1)$ represents the Fermi energy around $-80\ meV(+50\ meV)$. As we can see, the DMI of Bloch-type spin structures is larger than that of Néel-type spin structures. Besides, the DMI of Bloch-type spin structures in Fig. S12 highly depends on the Fermi energy, this is qualitatively consistent with our experimental observation in Fig. 3c (shifting the Fermi energy can largely increase the topological Hall resistivities). However, the DMI of Néel-type spin structures is almost independent of the Fermi energy. Generally speaking, Néel-type spin structures are usually observed in interface system or surface (ultra-thin film), while a Bloch-type spin structures are more likely to be observed in chiral bulk magnets. Thus, combining both theoretical analysis and experimental observations, we prefer a two-dimensional (2D) Bloch-type spin textures.

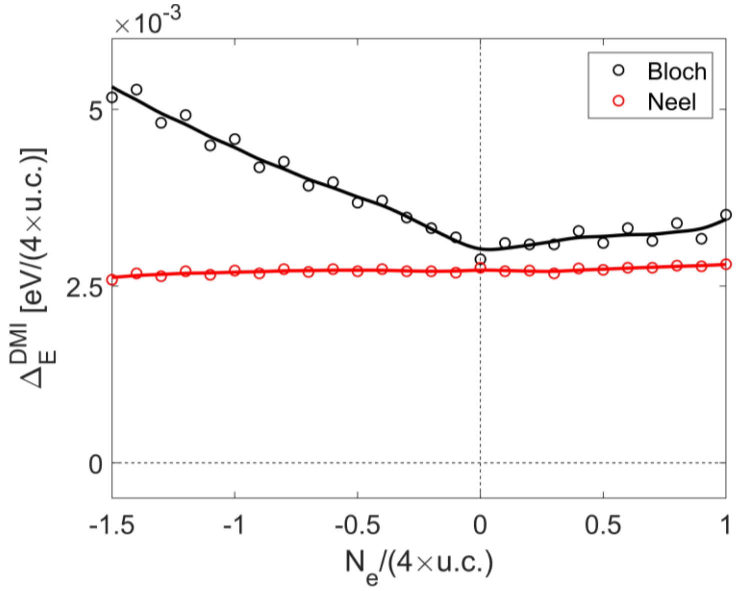

Figure S12. Comparison of DMI between Bloch-type and Néel-type spin structures. The electron number N_e dependent DMI strength $\mathbf{d}_{\perp}$ ($\propto \Delta_E^{DMI}$) for both Bloch-type and Néel-type spin structures. $N_e = 0$ represents the case of $E_F = 0$, and $N_e = -1.5(+1)$ represents the Fermi energy around $-80\ meV(+50\ meV)$.

14. Comparison of THE to other compounds.

Table 1 shows the maximal magnitude of THE observed in different systems. The maximal amplitude of THE observed in van der Waals (vdW) magnet Fe_xTaS_2 reaches 1.4 $\mu\Omega \cdot cm$, the same order as recently observed giant THE in Frustrated triangular-lattice Gd_3PdSi_3 [24]. This huge THE is larger than most of the known magnetic materials so far. The huge THE and anomalous Hall effect observed in our experiments indicates that vdW magnet Fe_xTaS_2 compound is a promising platform harbouring nontrivial spin textures associated with the Berry curvatures in both real and momentum spaces.

Table 1. The maximal THE observed in various compounds.

Compounds	Maximal THE ($\mu\Omega \cdot cm$)	Notes	Refs
MnSi	0.0045	Chiral magnet	Ref. 18
Mn_5Si_3	0.05	Chiral magnet	Ref. 19
MnGe	0.16	Chiral magnet	Ref. 20
$SrRuO_3$ monolayer	~0.25	Correlated oxide	Ref.21
$Fe_{0.7}Co_{0.3}Si$	0.82	Chiral magnet	Ref. 22
$SrRuO_3$-$SrIrO_3$ interface	1.1	Interface system	Ref. 23
$Fe_{0.28}TaS_2$	1.4	Chiral vdW magnet	This work
Gd_3PdSi_3	~2.5	Frustrated triangular-lattice	Ref. 24
EuO	12	Centrosymmetric oxide	Ref. 25
$Ca_{0.99}Ce_{0.01}MnO_3$	120	Correlated oxide	Ref. 26
Cr-doped (Bi, Sb)$_2$Te$_3$ layers	>1800	Quantum anomalous Hall system	Ref. 27

References

1. C. J. Brinker, G. W. Scherer, Sol-Gel science: The physics and chemistry of Sol-Gel Processing, (Academic Press, SanDiego, Califolia, USA, 1990).
2. M. Jo *et al.*, Gate-induced massive and reversible phase transition of VO_2 channels using solid-State proton electrolytes. *Adv. Funct. Mater*. **28**, 1802003 (2018).
3. G. Zheng *et al.*, Gate-Tuned Interlayer Coupling in van der Waals Ferromagnet Fe_3GeTe_2 Nanoflakes. *Phys. Rev. Lett.* **125**, 047202 (2020).
4. G. Kresse, J. Furthmuller, Efficient iterative schemes for ab initio total-energy calculations using a plane-wave basis set, *Phys. Rev. B* **54**, 11169 (1996).
5. G. Kresse, D. Joubert, From ultrasoft pseudopotentials to the projector augmented-wave method. *Phys. Rev. B* **59**, 1758 (1999).
6. J. P. Perdew, K. Burke, M. Ernzerhof, Generalized Gradient Approximation Made Simple. *Phys. Rev. Lett*. **77**, 3865 (1996).
7. Y. G. Yao *et al.*, First principles calculation of anomalous Hall conductivity in ferromagnetic bcc Fe. *Phys. Rev. Lett.* **92**, 037204 (2004).
8. A. A. Mostofi, J. R. Yates, Y.-S. Lee, I. Souza, D. Vanderbilt and N. Marzari, wannier90: A tool for obtaining maximally-localised Wannier functions. *Comput. Phys. Commun.* **178**, 685 (2008).
9. X. Wang, J. R. Yates, I. Souza, D. Vanderbilt, Ab initio calculation of the anomalous Hall conductivity by Wannier interpolation. *Phys. Rev. B* **74**, 195118 (2006).
10. V. I. Anisimov, J. Zaanen, O. K. Andersen, Band theory and Mott insulators: Hubbard U instead of Stoner I, *Phys. Rev. B* **44**, 943 (1991).
11. V. I. Anisimov, O. Gunnarsson, Density-functional calculation of effective Coulomb interactions in metals, *Phys. Rev. B* **43**, 7570 (1991).
12. Eibschütz, M. et al. Ferromagnetism in metallic $FexTaS_2$ (x~0.28). *Appl. Phys. Lett.* **27**, 464 (1975).

13. Eibschütz, M. et al. Ferromagnetism in metallic intercalated compounds Fe_xTaS_2 (0.20 < x <0.34). *J. Appl. Phys.* **52**, 2098 (1981).
14. Dijkstra, J., Zijlema, P. J., van Bruggen, C. F., Haas, C. & de Groot, R. A. Band-structure calculations of $Fe_{1/3}TaS_2$ and $Mn_{1/3}TaS_2$, and transport and magnetic properties of $Fe_{0.28}TaS_2$. *J. Phys.: Condens. Matter* **1**, 6363–6379 (1989).
15. Kim, K. et al. Large anomalous Hall current induced by topological nodal lines in a ferromagnetic van der Waals semimetal. *Nat. Mat.* 17, 794-799 (2018).
16. Groenendijk, D. J. et al. Berry phase engineering at oxide interfaces. *Phys. Rev. Res.* **2**, 023404 (2020).
17. Fijalkowski, K. M. et al. Coexistence of surface and bulk ferromagnetism mimics skyrmion Hall effect in a topological insulator. *Phys. Rev. X* **10**, 011012 (2020).
18. Neubauer, A. et al. Topological Hall Effect in the *A* Phase of MnSi. *Phys. Rev. Lett.* **102**, 186602 (2009).
19. Sürgers, C., Fischer, G., Winkel, P. & Löhneysen, H. v. Large topological Hall effect in the non-collinear phase of an antiferromagnet. *Nat. Commun.* **5**, 3400 (2014).
20. Kanazawa, N. et al. Large topological Hall effect in a short-period helimagnet MnGe. *Phys. Rev. Lett.* **106**, 156603 (2011).
21. Qin, Q. et al. Emergence of topological Hall effect in a $SrRuO_3$ single layer. *Adv. Mater.* **31**, 1807008 (2019).
22. Porter, N. A. et al. Giant topological Hall effect in strained $Fe_{0.7}Co_{0.3}Si$ epilayers. Preprint at https://arxiv.org/abs/1312.1722 (2013).
23. Matsuno, J. et al. Interface-driven topological Hall effect in $SrRuO_3$-$SrIrO_3$ bilayer. *Sci. Adv.* **2**, e1600304 (2016).
24. Kurumaji, T. et al. Skyrmion lattice with a giant topological Hall effect in a frustrated triangular-lattice magnet. *Science* **365**, 914–918 (2019).
25. Ohuchi, Y. et al. Topological Hall effect in thin films of the Heisenberg ferromagnet EuO. *Phys. Rev. B* **91**, 245115 (2015).

26. Vistoli, L. et al. Giant topological Hall effect in correlated oxide thin films. *Nat. Phys.* **15**, 67-72 (2019).

27. Jiang, J. et al. Concurrence of quantum anomalous Hall and topological Hall effects in magnetic topological insulator sandwich heterostructures. *Nat. Mat.* **19**, 732-737 (2020).
